# Influenza A Virus NS1 Protein Suppresses JNK1-Dependent Autophagosome Formation Mediated by Rab11a Recycling Endosomes

**DOI:** 10.3389/fmicb.2018.03120

**Published:** 2018-12-14

**Authors:** Takahiro Kuroki, Suguru Osari, Kyosuke Nagata, Atsushi Kawaguchi

**Affiliations:** ^1^Graduate School of Comprehensive Human Sciences, University of Tsukuba, Tsukuba, Japan; ^2^Department of Infection Biology, Faculty of Medicine, University of Tsukuba, Tsukuba, Japan; ^3^Transborder Medical Research Center, University of Tsukuba, Tsukuba, Japan

**Keywords:** autophagosome, influenza virus, JNK1, NS1, recycling endosome

## Abstract

Autophagy is an essential process for cellular metabolism and homeostasis, but also functions as one of innate immune responses against pathogen infection. However, in contrast to cellular metabolism and homeostasis pathways, less is known about how virus infection leads to autophagosome formation. Here, we showed that influenza A virus NS1 protein inhibits the formation of autophagosomes. The autophagosome formation was induced by infection with NS1 mutant virus lacking the dsRNA-binding activity for inhibition of innate immune responses (R38AK41A) or the activation of PI3K-Akt signaling pathway (Y89F). R38AK41A mutant infection induced phosphorylation of JNK1 and up-regulated the expression of autophagy-related genes which are downstream of JNK1 signaling pathway. We also found that the amount of phosphorylated TSC2, which activates mTOR, increased in wild type-infected cells but not in Y89F mutant-infected cells. These findings suggest that NS1 inhibits the autophagosome formation through both the inhibition of JNK1 and the activation of PI3K-Akt-mTOR pathway. Further, viral ribonucleoprotein (vRNP) complexes were selectively sequestered into autophagosomes, and knockdown of Rab11a, which is responsible for the apical transport of vRNP complexes, impaired not only engulfment of vRNP complexes by autophagosomes but also the formation of autophagosomes in R38AK41A mutant-infected cells. This indicates that Rab11a-positive recycling endosomes function as a donor membrane for the phagophore elongation and an autophagic receptor for the selective engulfment of viral RNP complexes. Based on these results, we propose that NS1 inhibits JNK1-mediated autophagy induction and the sequestration of vRNP complexes into autophagosomes.

## Introduction

Autophagosome is a cytoplasmic organelle and consists of double-membrane vesicles that contain parts of the cytoplasm and organelles. In response to various stimuli, including nutrient starvation, organelle damages, and pathogen infection, cytosolic materials are sequestered by expanding isolated membrane via activation of Beclin-1/Vps34 complex followed by LC3 processing (Kabeya et al., 2000; Funderburk et al., 2010). The constituents are eliminated from cells through either the degradative autophagy pathway by fusing autophagosomes with lysosomes or the secretory autophagy pathway mediated by multivesicular bodies (MVB; Fader and Colombo, 2009). The selective autophagy of pathogens including viruses, bacteria, and protozoan is termed xenophagy (Levine, 2005; Bauckman et al., 2015), and xenophagy plays an important role in innate immunity by promoting entrapment and degradation of a broad range of intracellular pathogens.

The mTOR pathway is one of the most evolutionary conserved autophagic pathway. mTOR complex 1 (mTORC1) is assembled when cellular nutrients are not limiting, and mTORC1 negatively regulates the kinase activity of ULK1 complex which plays a key role in the autophagy induction (Kamada et al., 2000; Hosokawa et al., 2009). Upon several stresses, including ER stress, starvation, and ROS production, a stress-activated signaling kinase, c-Jun N-terminal protein kinase 1 (JNK1) also regulates the autophagy induction by phosphorylating Bcl-2 that disrupts the Bcl-2/Beclin-1 complex for the assembly of Beclin-1/Vps34 complex (Wei et al., 2008; Cheng et al., 2014; Zhong et al., 2017). In addition to these post-translational regulations, autophagy is also regulated by transcription of autophagy-related genes (ATG genes). It is reported that JNK1 activation is necessary for up-regulation of Beclin-1 expression through the phosphorylation of transcription factor c-Jun (Li et al., 2009). Not only AP-1 family members, including c-Jun and c-Fos, but also FoxO transcription factors are regulated by JNK1, and the phosphorylated FoxO proteins induce the expression of multiple ATG genes, including *ATG12*, *Bnip3*, *LC3B*, and *Ulk2* genes (Mammucari et al., 2007; Zhao et al., 2007, 2008).

In contrast to the autophagic pathways for cellular homeostasis, less is known about how virus infection leads to the autophagosome formation. Pathogen-associated molecular patterns (PAMPs) are essential components derived from infectious pathogens to distinguish self from non-self and to promote the immune responses. PAMPs, such as viral RNAs and bacterial components, are recognized by pathogen recognition receptors (PRRs), such as Toll-like receptors (TLRs), NOD-like receptors (NLRs), RIG-I-like receptors (RLRs), and double-stranded RNA-binding protein kinase PKR (Takeuchi and Akira, 2010). Several PRRs, including TLR3, RIG-I, and PKR, recognize intracellular dsRNA which is a common byproduct of viral replication as a PAMP. It is reported that autophagy is induced through TLR7 signaling triggered by ssRNA (Delgado et al., 2008) and PKR-mediated phosphorylation of eIF2α upon HSV-1 infection (Talloczy et al., 2006). However, the exact mechanism of autophagy induction by influenza virus infection is unclear.

The autophagic degradation of viral components is also known to promote innate immunity through delivery of viral PAMPs to TLR in the endosomes and adaptive immunity by feeding antigens to MHC class II compartments (Paludan et al., 2005; Blanchet et al., 2010). Given these integral roles of autophagy in antiviral immune responses, it is believed that viruses have developed specific strategies to counteract autophagy. Influenza A virus (IAV) is an enveloped virus with eight-segmented and single-stranded genomic RNAs of negative polarity (McCauley and Mahy, 1983). The viral genome exists as viral ribonucleoprotein (vRNP) complexes by interacting with nucleoprotein (NP) and viral polymerase complex consisting of PB1, PB2, and PA, and is transported to the apical plasma membrane through Rab11a-positive recycling endosomes for virus budding. It is reported that IAV infection induces the autophagosome formation in cells constitutively expressing GFP-LC3B, but the fusion of autophagosomes with lysosomes is inhibited by viral M2 protein, which functions as a proton ion channel (Gannage et al., 2009). Further, the over-expressed HA glycoprotein and M2 are thought to induce the lipidation of LC3 in transfected cells (Zhirnov and Klenk, 2013). However, the exact mechanism of autophagosome activation by IAV infection and its inhibition by viral protein(s) remains unclear.

Here, we showed that viral NS1 protein suppresses JNK1-mediated autophagosome formation. NS1 is composed of N-terminal dsRNA binding domain and C-terminal effector domain, and plays a major role in the inhibition of cellular innate immune responses (Hale et al., 2008). NS1 inhibits the activation of antiviral proteins, including RIG-I, PKR, and 2′-5′-oligoadenylate synthetase by competing with them for dsRNA (Hatada et al., 1999; Bergmann et al., 2000; Min and Krug, 2006; Guo et al., 2007; Rehwinkel et al., 2010). We found that the autophagosome formation was induced by infection with NS1 mutant virus (R38AK41A) lacking the dsRNA binding activity. The formation of autophagosomes in R38AK41A-infected cells was dependent on the JNK1 activation. Further, vRNP complexes were selectively recruited to autophagosomes through Rab11a-positive recycling endosomes in R38AK41A-infected cells. These results suggest that dsRNA-mediated antiviral signaling pathway activates JNK1 to induce autophagosome formation, and then Rab11a-positive recycling endosomes function as an autophagic receptor for the selective engulfment of viral RNP by autophagosomes. Collectively, we propose that NS1 inhibits the JNK1 activation for autophagy induction and the sequestration of vRNP complexes into autophagosomes to enable proper endocytic transport to the plasma membrane.

## Results

### The Influenza A Virus NS1 Protein Inhibits the Autophagosome Formation

**FIGURE 1:**
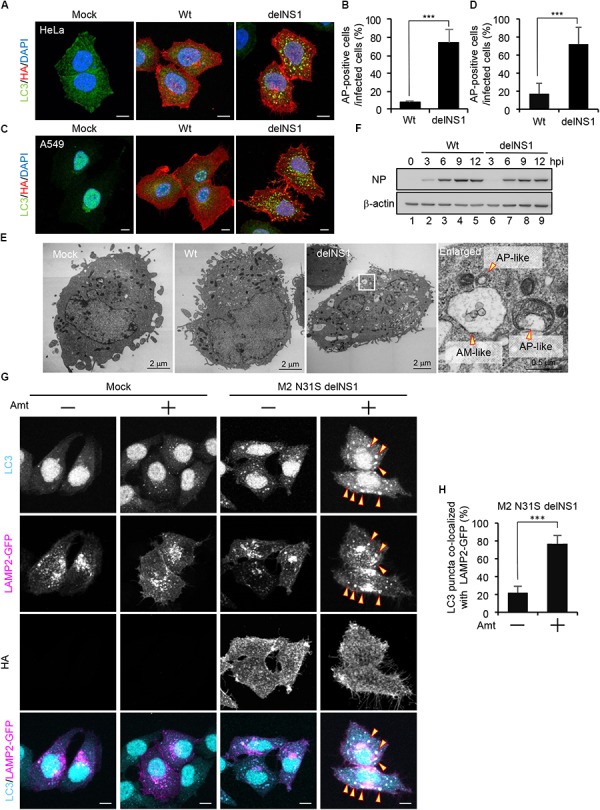
The influenza A virus NS1 protein inhibits the autophagosome formation. **(A–D)** HeLa cells **(A)** and A549 cells **(C)** were infected with influenza A/PR/8/34 virus (Wt) or delNS1 virus at MOI of 3. At 10 h post-infection, cells were subjected to the indirect immunofluorescence assays with mouse anti-HA (red) and rabbit(anti-LC3 antibodies (green). Nuclei were stained with DAPI (blue). Scale bar, 10 μm. The average percentage of cells exhibiting LC3 puncta relative to total infected cells and standard deviations determined from three independent experiments were shown in panel **(B)** (HeLa cells) and **(D)** (A549 cells) (AP; Autophagosome, *n* > 100). The statistical significance was determined by Student’s *t*-test, ^∗∗∗^*P* < 0.001. **(E)** HeLa cells were infected with delNS1 virus at MOI of 3. At 10 h post-infection, cells were pelleted and subjected to transmission electron microscopy analysis (AP; Autopahgosome, AM; Amphisome). **(F)** HeLa cells were infected with either Wt (lanes 2–5) or delNS1 (lanes 6–9) virus at MOI of 3. At 3, 6, 9, and 12 h post-infection, the cells were lysed, and the lysates were analyzed by SDS-PAGE followed by western blotting with anti-NP and anti-β-actin antibodies. **(G,H)** HeLa cells expressing GFP-LAMP2 (shown in magenta) were infected with M2-N31S delNS1 virus at MOI of 3 with or without 50 μM amantadine (Amt). At 10 h post-infection, cells were subjected to the indirect immunofluorescence assays with anti-LC3 (cyan) and anti-HA antibodies. The arrowheads indicate LC3 puncta co-localized with LAMP2-GFP. Scale bar, 10 μm. The average percentage of LC3 puncta co-localized with LAMP2-GFP relative to total LC3 puncta and standard deviations determined from three independent experiments were shown in panel **(H)** (*n* > 100). The statistical significance was determined by Student’s *t*-test, ^∗∗∗^*P* < 0.001.)

Previous studies reported that autophagosomes are formed upon IAV infection in GFP-LC3-expressing cells (Gannage et al., 2009; Zhou et al., 2009; Beale et al., 2014). However, we could not observe the autophagosome formation in wild-type IAV-infected HeLa cells (Figures 1A,B) and A549 cells (Figures 1C,D) without stable expression of GFP-LC3. In contrast, we found that LC3 accumulated in cytoplasmic punctate structures upon infection of delNS1 mutant virus which contains a deletion of *NS1* gene, indicating that NS1 represses the formation of autophagosomes in these cells (Figures 1A–D). Further, typical autophagosome-like vacuoles (AP) consisting of double-membrane vesicles and amphisome-like structures (AM), which are possibly generated by membrane fusion between autophagosomes and endosomes, were observed in delNS1-infected cells by transmission electron microscopy (TEM) analysis, but not in mock-treated cells and wild-type-infected cells (Figure 1E). However, the amount of NP protein was not reduced in delNS1-infected cells compared with that of wild-type virus (Figure 1F). Previous studies revealed that M2 inhibits the fusion of autophagosomes with lysosomes that is a necessary process for autophagic degradation (Gannage et al., 2009). In agreement with previous reports, LC3 puncta were hardly colocalized with GFP-LAMP2, a lysosomal marker, in amantadine sensitive M2-N31S delNS1-infected cells (Figure 1G). In contrast, by adding 50 μM amantadine, a potent inhibitor of M2 ion channel activity, LAMP2 was colocalized with autophagosomes in M2-N31S delNS1-infected cells (Figures 1G,H). These results indicate that IAV inhibits the autophagic pathway through the inhibition of autophagosome formation by NS1 and preventing the autophagosome fusion with lysosomes by M2.

### NS1 Suppresses the Autophagosome Formation Through the dsRNA-Binding and the PI3K Activating Activities

**FIGURE 2:**
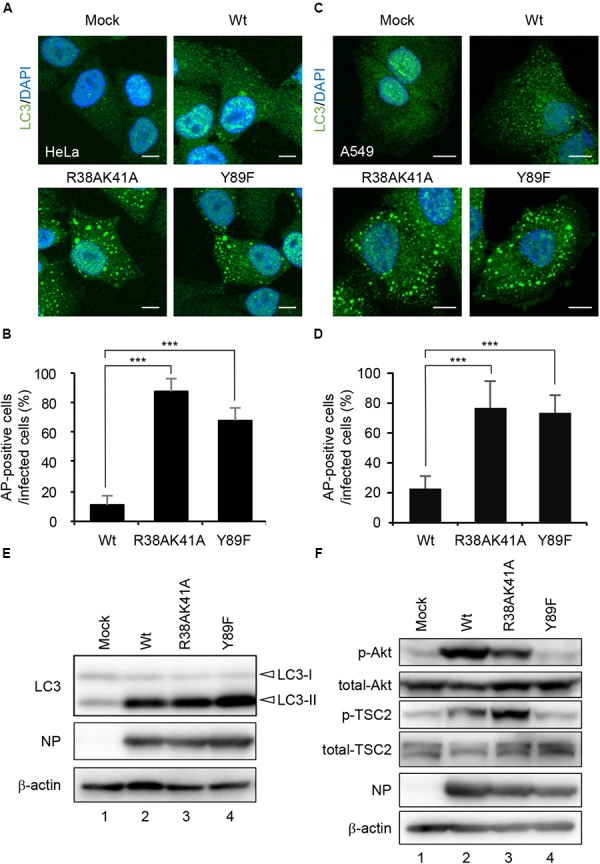
NS1 suppresses the autophagosome formation through both the dsRNA-binding activity and the activation of PI3K-Akt signaling pathway. **(A–D)** HeLa cells **(A)** and A549 cells **(C)** were infected with either Wt, R38AK41A, or Y89F virus at MOI of 3. At 10 h post-infection, cells were subjected to the indirect immunofluorescence assays with anti-LC3 antibody (green). Nuclei were stained with DAPI (blue). Scale bar, 10 μm. The average percentage of cells exhibiting LC3 puncta relative to total infected cells and standard deviations determined from three independent experiments were shown in panel **(B)** (HeLa cells) and **(D)** (A549 cells) (AP; Autophagosome, *n* > 100). The statistical significance was determined by Student’s *t*-test, ^∗∗∗^*P* < 0.001. **(E)** HeLa cells were infected with Wt, R38AK41A, or Y89F virus at MOI of 3. At 20 h post-infection, the cell lysates were prepared and subjected to western blotting assays with anti-LC3B, anti-NP, and anti-β-actin antibodies. **(F)** HeLa cells were infected with Wt, R38AK41A or Y89F virus at MOI of 3. At 4 h post-infection, the cell lysates were prepared and subjected to western blotting assays with anti-phospho-Akt, anti-Akt, anti-phospho-TSC2, anti-TSC2, anti-NP, and anti-β-actin antibodies.

NS1 is a multifunctional dsRNA-binding protein and suppresses a number of cellular antiviral activities. The dsRNA-binding activity of NS1 is required for the inhibition of host innate immune responses by competing with antiviral proteins for dsRNA (Hatada et al., 1999; Bergmann et al., 2000; Min and Krug, 2006; Guo et al., 2007; Rehwinkel et al., 2010). NS1 also interacts with regulatory subunit p85β of class I PI3K (Hale et al., 2006) and activates the PI3K-Akt pathway, which inhibits the autophagosome formation via activation of mTORC1 (Hale et al., 2006; Shin et al., 2007). To address the molecular mechanism of autophagy inhibition by NS1, HeLa cells (Figures 2A,B) and A549 cells (Figures 2C,D) were infected with either R38AK41A mutant deficient in the dsRNA-binding activity or Y89F mutant deficient in the stimulatory activity of class I PI3K (Wang et al., 1999; Hale et al., 2006), and then cells were subjected to indirect immunofluorescence assays with anti-LC3 antibody at 10 h post-infection. In contrast to wild-type virus, LC3 puncta were formed in R38AK41A- and Y89F-infected cells (Figures 2A–D). The level of LC3 lipidation, in which cleaved LC3 (LC3-I) is conjugated with phosphatidylethanolamine (PE) to become a membrane-bound form (LC3-II), is known to correlate with the extent of autophagosome formation (Kabeya et al., 2000). The LC3 lipidation was induced by wild-type-infected cells as previously reported (Zhirnov and Klenk, 2013), and the extent was not up-regulated in R38AK41A and Y89F-infected cells (Figure 2E). This indicates that NS1 inhibits the autophagosome formation in a LC3 lipidation-independent manner.

The PI3K-Akt signaling pathway is known to activate mTORC1 through the phosphorylation of TSC2, and the activated mTORC1 inhibits the ULK1/2-ATG13-FIP200 complex-dependent phagophore formation (Backer, 2008; Hosokawa et al., 2009; Huang and Manning, 2009). We found that the amount of phosphorylated TSC2 increased in wild type-infected cells (Figure 2F, lane 2) but not in Y89F-infected cells (Figure 2F, lane 4) at 4 h post-infection. This suggests that NS1 stimulates the PI3K-Akt-mTOR pathway to counteract the autophagosome induction upon IAV infection. In contrast, autophagosomes were induced in R38AK41A-infected cells (Figures 2A,B) even in the presence of phosphorylated TSC2 (Figure 2F, lane 3). These results indicate that the activation of PI3K-Akt signaling pathway by NS1 is not sufficient to inhibit the autophagosome formation, and another pathway also needs to be inhibited by NS1 through the dsRNA-binding activity.

### JNK1 Activation Is Required for the Autophagosome Formation in R38AK41A Mutant-Infected Cells

**FIGURE 3:**
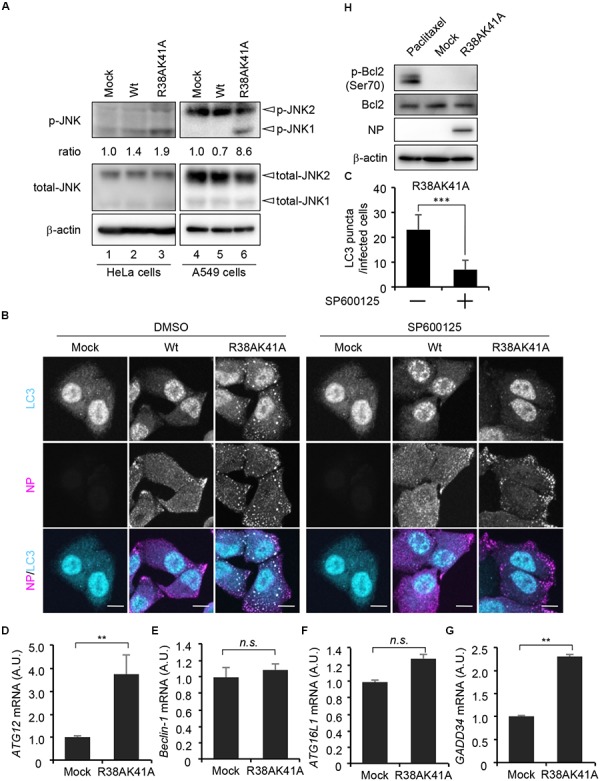
JNK1 signaling pathway is required for the autophagosome formation in R38AK41A-infected cells. **(A)** HeLa cells were infected with Wt or R38AK41A virus at MOI of 3. At 4 h post-infection, cell lysates were subjected to western blotting assays with anti-phospho-JNK, anti-JNK, anti-NP, and anti-β-actin antibodies (lanes 1–3). The p-JNK/β-actin ratios determined by FUSION system (Vilber-Lourmat) are shown underneath the p-JNK blot. The JNK1 activation in A549 cells was also examined (lanes 4–6). **(B,C)** HeLa cells were infected with either Wt or R38AK41A virus at MOI of 3. At 4 h post-infection, cells were treated for 6 h with SP600125, a JNK inhibitor, and then subjected to the indirect immunofluorescence assays with anti-LC3 (cyan) and anti-NP (magenta) antibodies. Scale bar, 10 μm. The average number of LC3 puncta in R38AK41A-infected cells treated with or without SP600125 and standard deviations determined from three independent(experiments were shown in panel C (*n* > 100). The statistical significance was determined by Student’s *t*-test, ^∗∗∗^*P* < 0.001. **(D–G)** HeLa cells were infected with R38AK41A virus at MOI of 3. At 4 h post-infection, total RNAs were isolated and subjected to quantitative RT-PCR with primer sets specific for *ATG12* **(C)**, *Beclin-1* **(D)**, *ATG16L1* **(E)**, and *GADD34* **(F)** mRNAs. The mean value and standard deviations obtained from three independent experiments are shown. ^∗∗^*P* < 0.01 by Student’s *t*-test. **(H)** HeLa cells were infected with R38AK41A virus at MOI of 3. At 4 h post-infection, the cell lysates were subjected to western blotting assays with anti-phospho-Bcl-2, anti-Bcl-2, anti-NP, and anti-β-actin antibodies. HeLa cells were treated with 10 nM paclitaxel for 12 h as a positive control for phosphorylated Bcl-2 (lane 1).)

Autophagosome formation is regulated by not only the TSC-mTOR pathway but also JNK1, which activates phagophore formation and elongation through transcriptional and post-transcriptional regulations. We next examined whether JNK1 signaling pathway is implicated in the autophagosome formation upon R38AK41A mutant infection by western blot assays with anti-phospho-JNK antibody. We found that JNK1 was phosphorylated in R38AK41A-infected cells both in HeLa cells and A549 cells (Figure 3A, lanes 3and 6). Further, LC3 puncta were hardly observed in R38AK41A-infected cells treated with 20 μM SP600125, a potent inhibitor of JNK (Figures 3B,C).

The induction of autophagy was thought to be primarily dependent on post-translational regulation. However, recent findings indicate that transcriptional networks (Lee et al., 2014; Seok et al., 2014) and histone modifications of ATG genes (Fullgrabe et al., 2014) are also required to initiate and maintain the autophagy processes. We next examined the transcription level of JNK1 downstream genes related to the autophagosome formation in R38AK41A-infected cells at 4 h post-infection. JNK1 phosphorylates a large number of target proteins, most of which are transcription factors, including AP-1 and FoxO families. ATG genes which we examined, and their upstream transcription factors are as shown below: *ATG12* by FoxO (Figure 3D); *Beclin-1* by c-Jun or ATF4 (Figure 3E); *ATG16L1* by ATF4 (Figure 3F). We found that the amount of d *ATG12* mRNA increased about 3.5-fold by R38AK41A infection but not *Beclin-1* mRNA. It is possible that JNK1-FoxO signaling pathway is responsible for the enhanced expression of ATG genes in R38AK41A-infected cells. Notably, it is reported that PKR-mediated phosphorylation of eIF2α induces a selective translation of ATF4 transcription factor which plays a crucial role in stress responses such as autophagy and ER stress (Harding et al., 2000; He and Klionsky, 2009; B’chir et al., 2013). However, the transcription of *Beclin-1* and *ATG16L1* genes was not up-regulated in R38AK41A-infected cells, although *GADD34* gene, an ER stress-related gene but not ATG gene, increased possibly by ATF4 expression (Figure 3G). It has been known that *Beclin-1* and *ATG16L1* genes are essential for autophagy induction (Mizushima et al., 2003; Funderburk et al., 2010), it is possible that these genes are stably expressed by other transcription factors in HeLa cells. Further, it is reported that, upon phosphorylation of Bcl-2 by JNK1, Beclin-1 dissociates from Bcl-2 and regulates the lipid kinase Vps34 to initiate the phagophore formation (Pattingre et al., 2005; Wei et al., 2008). However, enhanced Bcl-2 phosphorylation at Ser70 was not observed in R38AK41A-infected HeLa cells at 4 h post-infection (Figure 3H).

### Viral RNP Complexes Are Selectively Engulfed by Autophagosomes Through Rab11a-Positive Recycling Endosomes

**FIGURE 4:**
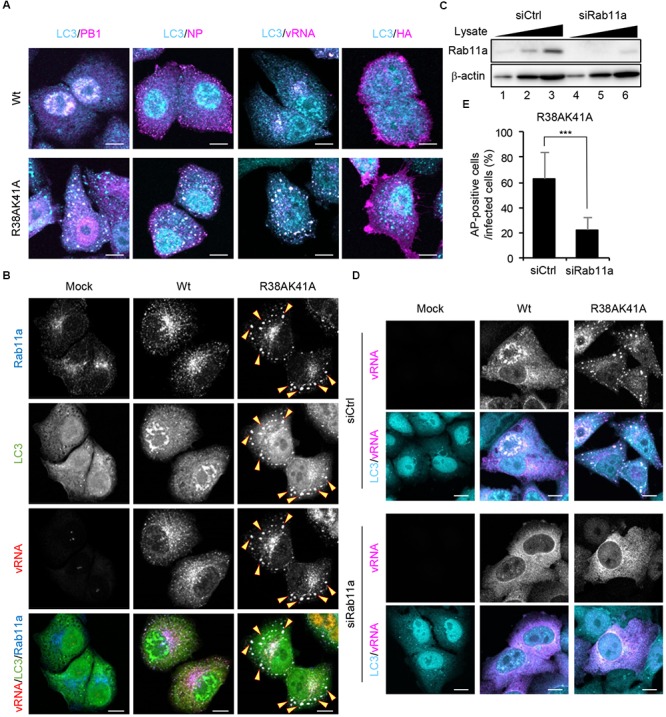
Viral RNP components are selectively engulfed by autophagosomes through Rab11a-positive recycling endosomes. **(A)** HeLa cells were infected with either Wt or R38AK41A virus at MOI of 3. At 10 h post-infection, cells were subjected to the indirect immunofluorescence assays with either anti-LC3 (cyan), anti-PB1 (magenta), anti-NP (magenta), or anti-HA antibodies (magenta), and then FISH assays to visualize the virus genome (magenta). Scale bar, 10 μm. **(B)** HeLa cells were infected with either Wt or R38AK41A virus at MOI of 3. At 10 h post-infection, cells were subjected to the indirect immunofluorescence assays with anti-LC3 (green) and anti-Rab11a antibodies (blue), and FISH assays to visualize the virus genome using a probe that hybridizes with segment 1 vRNA (red). The arrowheads indicate LC3 puncta stained with Rab11a and vRNA. Scale bar, 10 μm. **(C)** HeLa cells were transfected with either non-targeting or Rab11a siRNA. At 48 h post transfection, the cell lysates (5 × 10^3^, 1 × 10^4^, and 2 × 10^4^ cells) were analyzed by SDS-PAGE followed by western blotting assays with anti-Rab11a and anti-β-actin antibodies. **(D,E)** Control and Rab11a KD cells were infected with either Wt or R38AK41A virus at MOI of 3. At 10 h post-infection, cells were subjected to the indirect immunofluorescence assays with anti-LC3 (cyan) antibody and FISH assays using the probe that hybridizes with segment 1 vRNA to visualize the virus genome (magenta). Scale bar, 10 μm. The average percentage of cells exhibiting LC3 puncta relative to total infected cells and standard deviations determined from three independent experiments were shown in panel **(E)** (AP; Autophagosome, *n* > 100). The statistical significance was determined by Student’s *t*-test, ^∗∗∗^*P* < 0.001.

To examine which viral factors are sequestered into autophagosomes, wild-type or R38AK41A-infected cells were subjected to indirect immunofluorescence assays with anti-PB1, anti-NP, and anti-HA antibodies, and fluorescence *in situ* hybridization (FISH) assays with an RNA probe complementary to the segment 1 viral genome. We found that viral genome, PB1, and NP, but not HA, were colocalized with LC3 puncta in R38AK41A-infected cells (Figure 4A), suggesting that the components of vRNP complexes are selectively sequestered into autophagosomes. The progeny vRNP complexes are known to be transported to the plasma membrane through Rab11a-positive recycling endosomes by the interaction between Rab11a and vRNP complexes (Amorim et al., 2011; Eisfeld et al., 2011; Momose et al., 2011). Rab11a was also colocalized with LC3 in R38AK41A-infected cells but not wild type-infected cells (Figure 4B).

The autophagosome formation begins with the formation of phagophore and then the phagophore membrane expands to engulf autophagic cargo into autophagosomes. Although the origin of the membranes recruited to phagophore is still controversial, several cellular membrane compartments including the ER, Golgi, Rab11a-positive recycling endosomes, plasma membrane, and mitochondria are thought to be a supplier of membranes (Lamb et al., 2013). To elucidate whether vRNP is recruited to autophagosomes via Rab11a-positive recycling endosomes, we examined the formation of autophagosomes in Rab11a KD cells. At 48 h post transfection of Rab11a siRNA, the expression level of Rab11a in KD cells decreased to about 25% of that in control cells (Figure 4C). We found that Rab11a KD impaired not only engulfment of vRNP by autophagosomes but also the autophagosome formation induced by IAV infection (Figures 4D,E). These results indicate that NS1 prevents the membrane supply and the selective sequestration of vRNP complexes into autophagosomes by Rab11a-positive recycling endosomes.

## Discussion

It is reported that the expression of M2 or HA glycoprotein stimulates LC3 lipidation by unknown mechanism (Zhirnov and Klenk, 2013), although the autophagosome formation was not observed (Figures 1A–D). M2 interacts with LC3 through a highly conserved LC3-interacting region (LIR) located in the cytoplasmic tail of M2. The interaction of M2 with LC3 stimulates LC3 lipidation in part, but this binding is essentially required for the translocation of LC3 to the plasma membrane for assembly of stable viral particles (Beale et al., 2014). In general, the amount of LC3-II is correlated to the activation of autophagy. In our study, even in the presence of high level LC3-II (Figure 2E), the formation of autophagosomes was suppressed in wild-type virus-infected cells through the dsRNA-binding and the PI3K-activating activities of NS1 (Figures 2A,C). NS1 is known to inhibit the dsRNA-dependent antiviral signaling pathways by counteracting antiviral proteins, such as PKR and RIG-I, through its dsRNA-binding activity. We found that the dsRNA-binding activity of NS1 is required to inhibit the JNK1-mediated autophagy upon IAV infection (Figures 2A,C, 3B). PKR is a serine/threonine protein kinase and is thought to recognize viral RNAs as a PAMP. The activated PKR lead to several stress responses mediated by JNK, p38 MAPK, NF-κB, and phosphorylation of eIF2α (Dabo and Meurs, 2012). It is also reported that RIG-I induces autophagy via RIG-I-MAVS-TRAF6 signaling pathway upon Sendai virus infection (Lee et al., 2018). TRAF6 is known to phosphorylate JNK in TGF-β signaling and type I IFN signaling pathways (Yamashita et al., 2008; Yoshida et al., 2008). Thus, it is possible that antiviral proteins, such as PKR and RIG-I, recognize viral RNAs to activate JNK1 for autophagosome formation. However, it is reported that NS1 proteins of certain IAV subtypes, including avian IAV, have an intrinsic function for JNK1 activation (Nacken et al., 2014). It is also proposed that the activation of JNK signaling pathway is required for the viral genome replication and viral protein synthesis in H5N1 IAV-infected cells (Zhang et al., 2016). To further understand the mechanism of JNK-mediated stress response against IAV infection, it may be required to analyze subtype-specific cellular responses focusing on the host range restriction.

The eliminations of damaged organelles, protein aggregates, and intracellular pathogens are highly selective processes which require cargo recognition by autophagic receptors. An autophagic receptor is defined by its ability to bridge cargo and autophagosomal membrane, leading to the engulfment of cargo by the autophagic membrane (Stolz et al., 2014). Our study revealed that Rab11a-positive recycling endosomes are required for the selective autophagy of vRNP complexes (Figure 4). Thus, Rab11a-positive recycling endosomes may function as not only the membrane supplier but also the autophagic receptor through the interaction between Rab11a and vRNP complexes. It is reported that WIPI2, which recruits ATG16L1 to form autophagosomes, interacts with Rab11a on the phosphatidylinositol 3-phosphate (PtdIns3P)-enriched endosome membranes to destine the membranes to become autophagosomes (Puri et al., 2018). It is also reported that SNX18 is responsible for the endocytic transport of ATG9 and ATG16L1 from recycling endosomes to phagophore through the interaction with Dynamin-2 (Soreng et al., 2018). Thus, it is possible that these adaptor proteins are recruited to Rab11a-positive recycling endosomes upon virus infection, possibly through the JNK1 signaling pathway, to switch the destination of recycling endosomes from the plasma membrane to autophagosomes. Our findings may contribute to understanding of the upstream signaling pathway of Rab11a-positive recycling endosomes to function as the autophagic receptor.

## Materials and Methods

### Biological Materials

Rabbit polyclonal antibodies against NP and PB1 were prepared as previously described (Kawaguchi et al., 2005; Kawaguchi et al., 2011). Rabbit polyclonal antibodies against LC3 (SIGMA; L7543), Akt (CST; 9272), phospho-Akt (Ser473) (CST; 9271), phospho-JNK (Thr186/Tyr185) (CST; 9251), rabbit monoclonal antibodies against β-actin (CST; 8547), phospho-Bcl-2 (Ser70) (CST; 2827), Bcl-2 (Epitomics; 1017-1), and mouse monoclonal antibodies against α-tubulin (Sigma; DM1A), TSC2 (Santa Cruz; sc-271314), phospho-TSC2 (Ser1798) (Santa Cruz; sc-293149), JNK (Santa Cruz; sc-7325), HA (TaKaRa; C179), Rab11a (BD; 47/Rab11) were purchased. HeLa cells and A549 cells were grown in Dulbecco’s minimal essential medium (DMEM) containing 10% bovine fetal calf serum and incubated at 37°C in 5% CO_2_. For the construction of plasmid expressing LAMP2-GFP, the cDNA of *LAMP2* was amplified from HeLa cDNAs with primers 5′-GCCAGCTAGCGCCGCCACCATGGTGTGCTTCCGCCTC-3′ and 5′-GCGTGCTAGCGCAAATTGCTCATATCCAG-3′, and was cloned into pEGFP-N1 plasmid.

### Viruses

Influenza A/Puerto Rico/8/34 (A/PR/8/34) virus was grown at 35.5°C for 48 h in allantoic sacs of 11-days-old embryonated eggs, and then the infected allantoic fluids were collected and stored at –80°C until use. A/PR/8/34 virus lacking the *NS1* gene (delNS1 virus) was a generous gift from Garcia-Sastre et al. (1998; Icahn School of Medicine at Mount Sinai). The delNS1 virus harboring an amantadine sensitive mutation, N31S, in M2 protein (M2-N31S del NS1), NS1-R38AK41A, and NS1-Y89F mutant viruses were generated by reverse genetics (Neumann et al., 1999) using co-culture of HEK293T cells with MDCK cells stably expressing NS1 protein. The viral titers were determined by focus-forming assays. Briefly, a confluent monolayer culture of MDCK cells on cover slips was infected with each virus, and then was fixed with 4% PFA for 10 min at 4 h post-infection. The coverslips were subjected to indirect immunofluorescence assays using rabbit anti-NP antibody. The number of infected cell foci was counted and viral titers were calculated as focus forming units (FFU). The viral titer of each virus was 2.0 × 10^8^ FFU/ml for wild-type PR8, 3.5 × 10^7^ FFU/ml for delNS1, 3.5 × 10^7^ FFU/ml for M2-N31S delNS1, 1.1 × 10^7^ FFU/ml for R38AK41A, 6.4 × 10^7^ FFU/ml for Y89F, respectively. All *in vivo* experiments were carried out according to the Guideline for Proper Conduct of Animal Experiments from Science Council of Japan. The protocols for animal experiments were approved by Animal Care and Use Committee of the University of Tsukuba.

### Intracellular Localization of Viral Proteins and Viral Genome

Indirect immunofluorescence assays and FISH assays were carried out as previously described (Jo et al., 2010). Briefly, cells were fixed with 1% PFA for 5 min and then pre-permeabilized with 0.01% digitonin in PBS for 5 min. After being washed with PBS, cells were fixed in 4% PFA for 10 min and permeabilized with 0.5% Triton X-100 in PBS for 5 min. After incubation in PBS containing 1% bovine serum albumin for 30 min, coverslips were incubated with each antibody for 1 h and then further incubated with Alexa Fluor 488- and 568-conjugated secondary antibodies, respectively (Life Technologies). FISH assays were performed after indirect immunofluorescence assays using an RNA probe complementary to the segment 1 virus genome. Images were acquired by a confocal laser scanning microscopy (LSM700; Carl Zeiss) using x63 Apochromat objective (NA = 1.4).

### Transmission Electron Microscopy (TEM)

Cell pellets were fixed with 2.5% glutaraldehyde overnight at 4°C. After further fixation with 1% OsO_4_ for 30 min at 4°C, sequential dehydrations with ethanol in a step-wise manner were carried out followed by propylene oxide treatment, and embedded in Epon. The obtained ultrathin sections were stained with uranyl acetate and lead citrate, and observed by TEM (JEOL; JEM-1400).

### Gene Silencing Mediated by siRNA

Short interfering RNAs (siRNAs) against *Rab11a* gene was purchased from Life Technologies. Cells (5 × 10^5^) were transfected with 15 pmol of siRNA using Lipofectamine RNAi Max (Life Technologies) according to the manufacturer’s protocol.

### RNA Analysis

The mRNA amounts of *ATG12*, *Beclin-1*, *ATG16L1*, and *GADD34* genes were examined by RT-qPCR. Purified total RNAs were reverse-transcribed with oligo(dT)_20_ primer, and subjected to quantitative PCR using FastStart SYBR Green (Roche) with following specific primer sets: 5′-GCAGCTTCCTACTTCAATTGCT-3′ and 5′-CCAGCAGGTTCCTCTGTTCC-3′ for *ATG12*; 5′-GAGCAAATGAATGAGGATGACA-3′ and 5′-CACTCTTCAGCTCATCATCCAG-3′ for *Beclin-1*; 5′-TGCCCTGCAGATCACTTTTAC-3′ and 5′-GAGTCGCTTAGTGGCTGCTC-3′ for *ATG16L1*; 5′-GGAGGCTGAAGACAGTGGAGGCCCTG-3′ and 5′-CCTCTAGGGACACTGGTTGCCTCTC-3′ for *GADD34*; 5′-AACGGCTACCACATCCAAGG-3′ and 5′-GGGAGTGGGTAATTTGCGC-3′ for 18S rRNA. The results were normalized to the level of 18S rRNA.

## Author Contributions

AK conceived and designed the experiments. TK, SO, and AK performed the experiments. TK, SO, KN, and AK analyzed the data, contributed reagents, materials, and analysis the tools, and wrote the manuscript.

## Conflict of Interest Statement

The authors declare that the research was conducted in the absence of any commercial or financial relationships that could be construed as a potential conflict of interest.
